# Characterization of a photon counting micro computed tomography laboratory setup

**DOI:** 10.1093/rpd/ncaf120

**Published:** 2026-03-13

**Authors:** My Celander, Robin Krüger, Niccolò Peruzzi, Marie-Louise Aurumskjöld, Martin Bech

**Affiliations:** Department of Medical Radiation Physics, Clinical Sciences Lund, Lund University, Barngatan 4, 22185 Lund, Sweden; Department of Medical Radiation Physics, Clinical Sciences Lund, Lund University, Barngatan 4, 22185 Lund, Sweden; Department of Medical Radiation Physics, Clinical Sciences Lund, Lund University, Barngatan 4, 22185 Lund, Sweden; Department of Haematology, Oncology and Radiation Physics, Skåne University Hospital, Entrégatan 7, 22185 Lund, Sweden; Department of Clinical Sciences Malmö, Lund University, Jan Waldenströms gata 35, 214 28 Malmö, Sweden; Department of Medical Radiation Physics, Clinical Sciences Lund, Lund University, Barngatan 4, 22185 Lund, Sweden

## Abstract

The imaging capabilities of an in-house X-ray setup for micro computed tomography, consisting of a photon-counting detector and a liquid target source, are reported here. The effect of the finite X-ray source spot size on the image resolution is measured using the slanted edge method at different sample-to-detector distances, and the energy response of the detector is probed using clinically relevant contrast agents. The results show that the spatial resolution is limited to $12$  $\mu$m at large sample magnification. The energy resolution of the detector could not be estimated based on the present experiment and data analysis. The method used to measure the spatial resolution could be useful for research groups wanting to characterize similar X-ray setups.

## Background

Photon Counting Detectors (PCDs) have entered the clinical world in the form of Photon Counting Computed Tomography (PCCT) modalities, where the first PCCT became commercially available in 2021 [1]. The most notable differences when compared to the Energy-Integrating Detectors (EIDs) used in conventional CT modalities lie in the capabilities of energy resolution and improved spatial resolution [1–3].

Typical materials for the PCDs are cadmium-telluride, cadmium-zinc-telluride, or silicon [4]. When an incoming X-ray photon enters the sensor material, it creates electron-hole pairs that drift toward pixelated anodes, where they are registered as pulses. The pulse height, after being reshaped, is proportional to the energy of the incoming X-ray photon. The pulses are only counted if they exceed a set energy threshold [4]. The most practical use for energy thresholding is to set the lowest energy threshold above the electrical and thermal noise, effectively suppressing electronic noise to zero, thus improving the Signal-to-Noise Ratio (SNR) [1].

Another powerful application of energy resolution is the ability to discriminate different elements due to their unique K-edges [5]. This technique is especially interesting when applied to clinical contrast agents such as gadoteric acid (gadolinium) and iohexol (iodine). Iodine-based contrast agents are instrumental when performing clinical X-ray imaging of the bowel and blood vessels, among others. Iodine-based contrast agents are not perfectly safe due to allergies or weakened kidney function among some patients. It is therefore of great interest to find alternatives such as gadolinium-based contrast agents, which are already clinically used in the Magnetic Resonance Imaging field [5].

The PCCT capabilities of higher resolution show many advantages in a clinical setting. It will open up new ways of diagnosing pathologies that are defined by small anatomical changes, such as e.g. fine lesions in the breast that could point to tumors [4]. The higher resolution also enables improved examinations of the fine structure in bone and investigation of blood vessels. Besides improved SNR, the energy thresholding also enables the PCCT to gather spectral information that can be used in the image reconstruction to further improve soft tissue contrast, which can improve e.g. liver imaging [6]. The clinical PCCT does not yet achieve micro-scale resolution.

A recent development in X-ray sources is the liquid-metal-jet target, which has been commercially available for only about ten years [7]. The principle of producing X-rays with liquid targets is similar to that of rotating anode X-ray sources, where the generated heat is distributed on a target wheel spinning at constant velocity [8]. The major advantage of a liquid target X-ray source is that the brightness of the source is up to 100 times higher when operated with a micro-focus electron beam. [9]. The liquid target X-ray sources are not limited by thermal effects, and can produce a stable X-ray spot size independent of increased power. However, with increased power, evaporated target material contaminates the vacuum chamber and builds up debris on the exit window of the source, affecting the overall X-ray flux [10].

A myriad of different micro-CT setups exists, with different purposes and components. Their role in pre-clinical studies has been most significant due to their small pixel size compared to clinical CT. An early implementation was in the investigation of bone structures, and now it is used for a wide variety of applications such as vascular imaging and imaging of internal organs [11]. There are several commercial alternatives for *in-vivo* and *ex-vivo* applications that utilize EIDs, while only a few commercial micro-CT vendors offer PCDs [12]. Commercial micro-CT is limited when it comes to samples of greater sizes. Micro-CT is also an important tool in material science, where bigger industrial cabinets are used. Here, it is used to investigate the structure of the material in terms of material properties, such as porosity or fractures [13]. These cabinets are optimized for hard materials and metals.

Our setup comprises a combination of a PCD with a liquid metal jet X-ray source, in a flexible in-house custom design with full flexibility in adjusting distances between source, sample, and detector. The field of view is limited by the detector panel size to samples of 150 mm diameter, with 75 $\mu$m pixel size. At highly magnifying geometry, the effective resolution is limited by the X-ray spot size. In this paper, we present our experimental results and method for calibrating the X-ray spot size in addition to the results of testing the energy resolution of an experimental setup for Photon Counting micro-CT, combining a liquid metal jet X-ray source (from Excillum AB) with a CdTe photon counting detector (from DECTRIS AG).

## Methodology

The X-ray micro-CT setup consists of an in-house built assembly placed on a 2 m long optical table for mechanical stability. The liquid metal jet X-ray source is fixed at one end of the table, whereas the sample manipulation tower can be translated along the optical axis as well as in the perpendicular plane. Further, a rotation stage allows for step-wise or continuous rotation of the sample while acquiring tomographic projections. A PCD is mounted on a separate translation stage, which can move the detector along the beam axis.

The X-ray source is a MetalJet D2+ $70 \mathrm{keV}$ (Excillum AB, Sweden) [14]. The liquid target of the source is an alloy liquid at room temperature, consisting of $68\%$ gallium, $22\%$ indium, and $10\%$ tin (ExAlloyI1). The accelerator voltage is variable ($40-70 \mathrm{kV}$) but was operated constantly at the maximum voltage of $70 \mathrm{kV}$. The electron beam spot shape is an ellipsoid with a 1:4 height-width ratio, but becomes symmetric when viewed from the X-ray exit window positioned at a $90$ degree angle with respect to the electron beam. The electron beam spot size was varied from $10\times 40$  $\mu$m$^{2}$ to $20\times 80$  $\mu$m$^{2}$. When changing the X-ray spot size to be smaller than $20\times 80$  $\mu$m$^{2}$, the power is software limited to avoid debris formation in the jet chamber [10].

The sample manipulation stage consists of a goniometer head (Huber GmbH, Germany) mounted on top of a rotational stage, which in turn is mounted upon two motorized linear translation stages (Owis GmbH, Germany). Translation of the sample along the optical axis of the X-ray beam allows for adjustment of the geometric magnification of the X-ray projection image. By horizontal translation perpendicular to the optical axis, the sample can be removed from the beam for the acquisition of flat reference images with an empty beam. Further, the PCD is mounted on motorized translation stage, but was kept at a fixed position throughout the experiments presented here.

The PCD is an Eiger 2R CdTe 1M-W (Dectris AG, Switzerland) [15] with pixel size $p=75\times 75$  $\mu$m$^{2}$ and a 1 mega-pixel sensor of $2068\times 512 \mathrm{pixels}$ ($155.1 \mathrm{mm}\times 38.4 \mathrm{mm}$). The 0.75 mm thick CdTe sensor has an absorption efficiency above 90% in the X-ray energy range $10-35 \mathrm{keV}$ and has two variable energy thresholds in the range $4-80 \mathrm{keV}$.

### Image resolution limitation

As the sample is located relatively close to the X-ray source and far from the X-ray detector, a large geometric magnification of the image can be achieved. This magnification is given by the Source-to-Sample Distance (SSD) and the Source-to-Detector Distance (SDD) as $M=\mathrm{SDD}/\mathrm{SSD}$, where $M$ is the magnification factor. Correspondingly, the effective pixel size $p_{\mathrm{eff}}$ is reduced by the magnification factor, $M$, relative to the actual pixel size $p$ as


1 \begin{eqnarray*} p_{\mathrm{eff}} = p\frac{\mathrm{SSD}}{\mathrm{SDD}}.\end{eqnarray*}

At sufficiently large geometric magnifications, the finite size of the electron beam spot defining the X-ray spot size will significantly affect the image by penumbral blurring. Hence, to evaluate the achievable resolution at different source settings, we set off to measure the penumbral blur as a function of X-ray spot size and geometric magnification.

To evaluate the penumbral blur effect, the Edge Spread Function (ESF) was measured using the slanted edge method [16]; the SDD was fixed at $1430 \mathrm{mm}$, and the SSD was varied between $35 \mathrm{mm}$ ($M=40.9$) and $225 \mathrm{mm}$ ($M=6.4$) in $5 \mathrm{mm}$ steps. A NanoXSpot test sample [17] with well-defined edge structures was imaged to probe the ESF. The tilt of the sample edge was set to $10^{\circ }$ using the goniometer. The exposure time was $1 \mathrm{s}$ and the lower energy threshold was set to $4.5 \mathrm{keV}$, reducing electrical and thermal noise. The slanted edge measurement was repeated for spot sizes $10$  $\mu$m, $15$  $\mu$m, and $20$  $\mu$m. The X-ray tube power was $131 \mathrm{W}$, $190 \mathrm{W}$, and $250 \mathrm{W}$, respectively. Flat field images were acquired for each distance using the same settings.

### Separation of contrast agents using energy resolution

Two clinically used contrast agents, gadoteric acid and iohexol, were used to test the energy resolution of the PCD. Clinically relevant concentrations of the contrast agents were imaged together with an isotonic saline solution, which acted as a reference liquid, see Table 1. The SDD was set to $600 \mathrm{mm}$ and the SSD was set to $360 \mathrm{mm}$. Two energy thresholds were set for each acquisition; the lower energy threshold and upper energy threshold were moved in $5 \mathrm{keV}$ steps with an exposure time of $300 \mathrm{s}$. The lower energy threshold range was set between $5 \mathrm{keV}$ and $55 \mathrm{keV}$ while the upper threshold was set between $10 \mathrm{keV}$ and $60 \mathrm{keV}$. Flat field images were acquired for each energy threshold using the same settings.

**Table 1:** The average transmission for selected contrast agents at different energies.

Sample Number	Substance	Quantity
I	Gadoteric acid	0.025 mg/ml
II	Gadoteric acid	0.05 mg/ml
III	Gadoteric acid	0.1 mg/ml
IV	Gadoteric acid	0.2 mg/ml
V	Gadoteric acid	0.4 mg/ml
VI	Gadoteric acid	1.0 mg/ml
VII	Gadoteric acid	2.0 mg/ml
VIII	Iodhexol, Saline solution	0.25 ml, 21 ml
IX	Saline solution	0.9% NaCl

Vertical lines denote the X-ray absorption energies for iodine (33.1 keV) and gadolinium (50.2 keV).

### Data acquisition and analysis

The interface used to interact and change settings for the X-ray source, sample stages, and detector, as well as data analysis, is handled by Python scripts (Python $\mathrm{v} 3.12.3)$ [18] in a similar manner to that previously reported for our nano-CT setup [19].

### Extraction of X-ray source spot size

**Figure 1:**
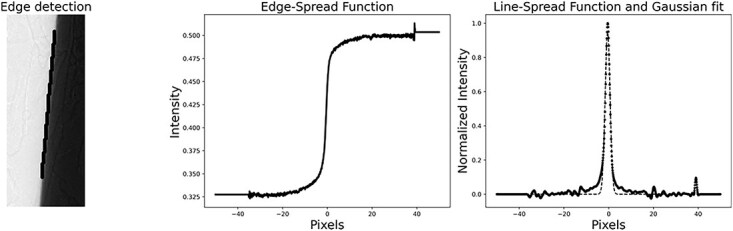
Left: the detected edge is marked by black data points, middle: plot of intensities in the vicinity of the edge (solid line), right: the LSF (dots) calculated from the ESF with included Gaussian fit (dashed lines).

A Region-Of-Interest (ROI) was set to define a sharp edge in the NanoXSpot test sample. A Gaussian filter was applied to create a smooth version of the edge image. The position of the edge inside the ROI could be determined as data points by subtracting the smoothed edge from the original edge. Outlier data points that appeared far from the border were removed by intensity thresholding, see the left part in Fig. 1.

The intensity values of the data points and the data point distance from the edge were extracted, and both 2D sets were flattened into 1D arrays. By binning and linear interpolation of the 1D data, an ESF was acquired. The difference between each adjacent point in the ESF was calculated, creating a Line Spread Function (LSF), and the result was fitted with a Gaussian function, and the Full Width Half Maximum (FWHM) could be calculated, see the middle and right parts of Fig. 1.

The LSF FWHM as a function of SSD represents the image blur at the detector, which depends on the penumbral blur, $w$, which can be expressed by the following equation:


2 \begin{eqnarray*}& w = s\frac{\mathrm{SDD}-\mathrm{SSD}}{\mathrm{SSD}}\end{eqnarray*}

where $s$ is the X-ray spot size and SDD and SSD are the Source-to-Detector Distance and Source-to-Sample Distance, respectively. At sufficiently large magnification, $w\gg s$ (and SDD $\gg$ SSD), and we can approximate Equation 2 into a linear dependence of SSD as


3 \begin{eqnarray*}& w \approx s\frac{\mathrm{SDD}}{\mathrm{SSD}} \Rightarrow \frac{1}{w} \approx \frac{\mathrm{SSD}}{\mathrm{SDD}\cdot s}.\end{eqnarray*}

The experimental X-ray spot size can be calculated from the penumbral blur by a linear fit of the measured $1/w_{\mathrm{FWHM}}$ as a function of SSD at high magnification (SSD = $35-75 \mathrm{mm}$). According to Equation 3, the resulting slope is $1/(\mathrm{SDD}\cdot s)$ and the FWHM of the source size $s$ can be extracted.

### Transmission spectra of contrast agents

When acquiring the transmission spectra of the different contrast agents, the dead pixels and detector gaps were inpainted using a biharmonic method [20]. X-ray transmission through the samples was calculated by normalization of the projection images by the flat image at the corresponding energy threshold. By subtracting the upper from the lower energy threshold measurement, an energy bin image with $5 \mathrm{keV}$ width is extracted. The image gray scale was adjusted to the highest concentration of gadoteric acid (vial VII).

**Figure 2:**
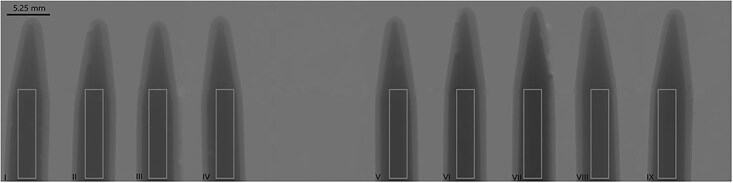
Regions of interest (ROIs) for calculating the average transmission through the different contrast agents.

A $50\times 250$ pixels ROI was placed over each vial containing contrast agent, see Fig. 2, and the pixel values were averaged for each ROI; this was repeated for each energy bin. The resulting average transmission is then plotted as a function of energy bin center.

## Results

### X-ray source spot size

**Figure 3:**
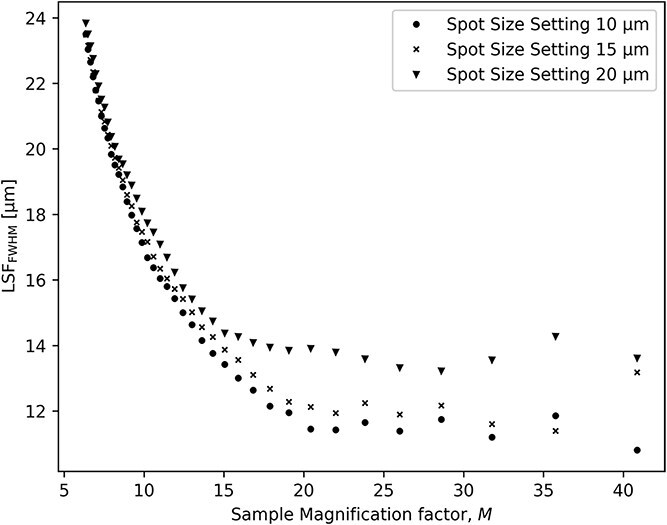
Image resolution at sample position as a function of sample magnification actor $M$. Note the plateau at large magnification.

**Figure 4:**
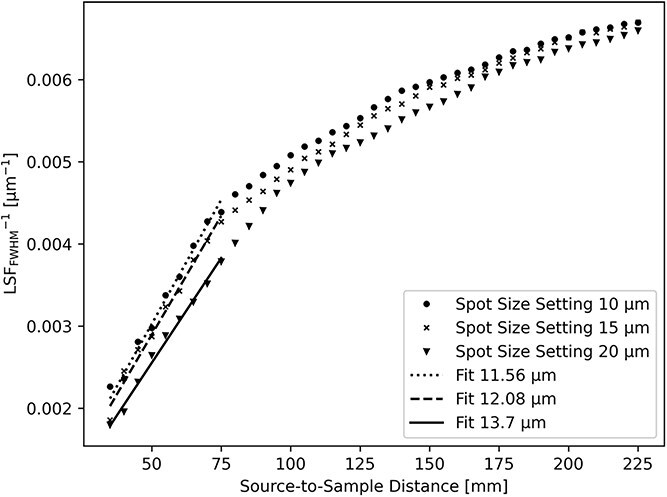
Plot of measured $1/w_{\mathrm{FWHM}}$ values as a function of SSD for the three different X-ray spot size settings shown together with corresponding linear fits at SSD $\leq 75$.

**Figure 5:**
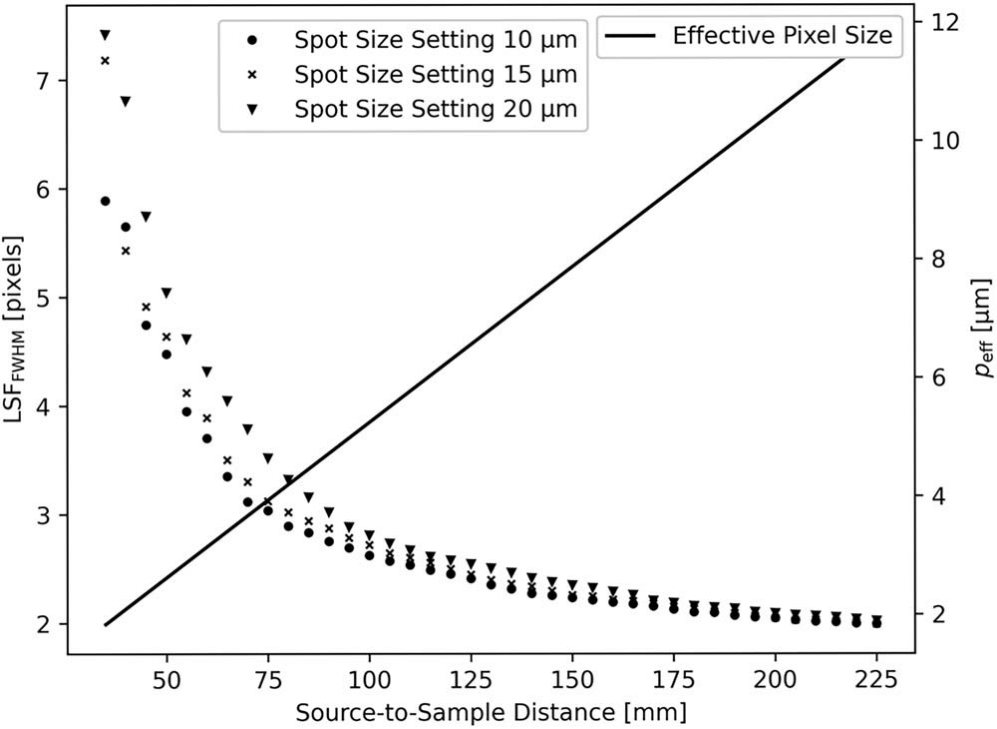
Left axis: Measured line-spread function (LSF$_{\mathrm{FWHM}}$) as a function of SSD for three different spot size settings. Right axis: Effective pixel size ($p_{\mathrm{eff}}$) calculated using Equation 1.

Figure 3 shows that at large magnification, there is a plateau where the resolution is independent of SSD and is hence limited by the penumbral blur. The size of the blur is extracted from the linear fit in Fig. 4. For a source spot size setting of $20$  $\mu$m, the resolution limit is $13.7$  $\mu$m for $M\geq 15.5$. For a source spot size setting of $15$$\mu$ m, the resolution limit is $12.1$  $\mu$m for $M\geq 19$. For a source spot size setting of $10$$\mu$ m, the resolution limit is only slightly improved to $11.6$  $\mu$m for $M\geq 19$. At lower magnifications ($M\leq 15$), which corresponds to SSD$\geq 95$ in Fig. 5, the blurring is less than three pixels.

### Energy resolution of contrast agents

**Figure 6:**
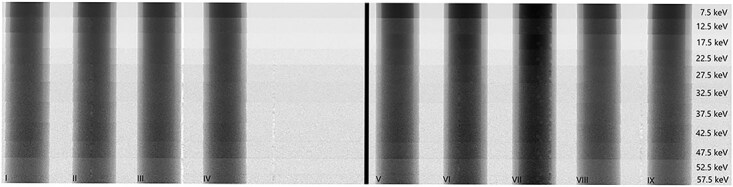
All eleven energy bin images stitched together for a visual comparison of the relative contrast, where the image gray scale is optimized for the gadolinium-based contrast agent (VII).

**Figure 7:**
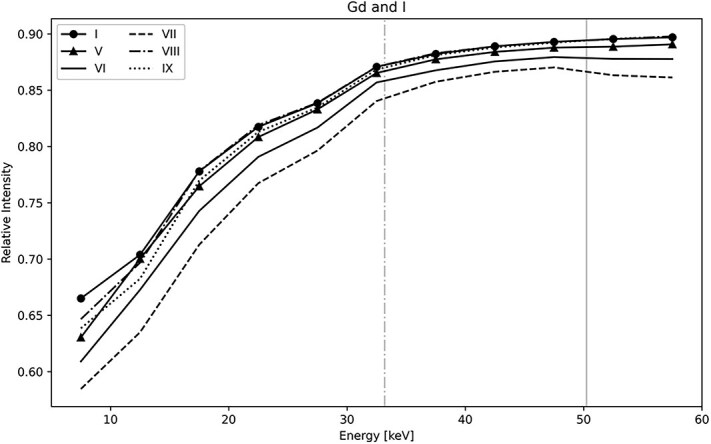
The average transmission for selected contrast agents at different energies, where vertical lines denote the X-ray absorption energies for iodine (33.1 keV) and gadolinium (50.2 keV).

The energy bin images displayed in Fig. 6, show the observed image contrast in gadoteric acid (vials I-VII) and iohexol (vial VIII). It can be seen that the clinically relevant concentrations of gaditric acid are very similar in contrast (vials I-V). The higher concentrations of gaditric acid stand out more (vials VI and VII). Each row in Fig. 6 denotes the energy bin centroid, and it can be seen that the observed contrast between each vial within one row is very similar. This behavior becomes more evident when the relative transmission through selected vials is plotted in Fig. 7.

## Discussion

The highest spatial resolution that can be achieved at our setup is limited to $\sim$12 $\mu$m at large sample magnification and at a source size setting of 15 $\mu$m where the power of the X-ray tube is limited to 190 W. Decreasing the X-ray spot size setting significantly reduces the tube power to 131 W with no significant improvement to the image resolution. Increasing the spot size setting to 20 $\mu$m significantly increases the power to 250 W while at the same time reducing the image resolution limit to $\sim$14 $\mu$m.

The current measurements of the X-ray spot sizes are dependent on the source’s current state. Slight variations can be expected when replacing components such as the electron cathode. Another source of uncertainty is the calibration of the electron beam spot size on the liquid anode. While the spot size remains stable during operation, a re-calibration may not reach the exact same spot size, but may differ by around $\pm 1$  $\mu$m. Another possible source of a systematic error could be the calibration of the SSD. However, comparing the theoretical magnification, which is dependent on the SSD, with an experimental magnification calculated from structures of known size in the NanoxSpot phantom, the discrepancies were small (below $1\%$ at small SSD where the structures were easily identified).

The image shown in Fig. 6 becomes increasingly noisier at higher energy bins. This could be combated by using larger energy bins, but at the cost of energy resolution. It could be of interest to redo the contrast agent measurement using an X-ray source with a higher acceleration voltage than 70 kV, where we speculate that higher counting statistics and less noise can be achieved in higher energy bins.

Due to the potential for energy differentiation in PCDs, we expected to see a difference in the transmission through gadolinium and iodine-based contrast agents, in particular in the vicinity of the K-edge absorption edges. However, no such difference was observed in our experiment, in contrast to the results previously reported by Badea *et al*. [21]. Badea *et al*. report a successful separation of different contrast agents, including iodine and gadolinium-based agents, using a setup which differed from ours mainly in the energy of the X-ray source. Their post-processing of the data involved an iterative reconstruction step implemented in an algebraic reconstruction. In our study, we compare only the radiographic contrast for simplicity, but we may implement a similar analytical approach in the future.

Our experimental setup was constructed to be flexible, allowing for a wide variety of samples to be imaged with a field-of-view ranging from a few millimeters to tens of centimeters. The detector has a relatively large sensor, which makes it possible to include full samples without reducing the magnification. The source can produce high flux, which allows for shorter scan times or improved SNR. Furthermore, the resolution can be adjusted freely, only limited by the physical size of the sample and the X-ray spot size. In contrast to the many different micro-CT configurations commercially available, our setup combines the advantages of a CdTe PCD and a liquid metal jet X-ray source. In theory, a liquid metal jet X-ray source could be implemented in a clinical setting for faster examination times due to higher flux; however, only in a radiographic setting with a horizontal beam. The liquid-based source can not be mounted on a rotating gantry, which unfeasible for tomography applications where the sample can not be rotated. This limitation makes liquid sources still only suitable for pre-clinical *ex vivo* purposes.

## Conclusion

It can be concluded that for flexible micro-CT systems like the one presented here, where magnification can be adjusted in a large range, an optimal geometry can easily be found by the measurements of the detected ESF. With our hardware, the best setting for high resolution (magnification $M\geq 15.5$) is the X-ray spot size setting $15$  $\mu$m. In situations where resolution requirements are more relaxed (magnification $M < 15.5$), the high power setting of X-ray spot size $20$  $\mu$m is preferred for improved X-ray flux. The method to achieve these results could be used to characterize other similar X-ray setups.

The PCD energy resolution capabilities, when it comes to distinguishing clinically relevant iodine and gadolinium-based contrast agents, yielded no significant result with the current experimental data and analysis.
